# Limitations in Treatment and Filling of Roots

**Published:** 1890-09

**Authors:** J. A. Bazin

**Affiliations:** Montreal, Canada


### LIMITATIONS IN TREATMENT AND FILLING OF ROOTS.[1]

BY DR. J. A. BAZIN, MONTREAL, CANADA.

THE numerous articles that have appeared in our dental journals for some time past upon "Root Treatment and Filling" have caused me to doubt my experience and observation, and also with great diffidence to question the statements and almost dogmatic assertions of some of the writers.

More recently has this been the case in reading an article and discussion reported in the INTERNATIONAL DENTAL JOURNAL by Professor James Truman, wherein he presents such a picture of almost complete perfection that I am forced to ask myself, as well as my associates, Can he have the same kind of teeth and the same order of beings to deal with as come into our hands? Believing that he has, and giving him, and all others who claim such full success in this branch of operations, credit for far greater deftness in manipulation and skill than I possess, yet the effect upon me has been to take up this subject for consideration and try and deal with it in the light of the, perhaps, limited experience and tests that I am able to offer. The first proposition I shall try to establish is that it is far from being commonly possible to extirpate all the contents of the pulp-canals of all the three-rooted teeth while in their normal position in the human jaws. Nature is exceedingly fickle in the anatomy of the roots of the teeth, and I might go further and say in all the details of the human frame, but will only deal, at this time, with one special subject. In proof, I exhibit a few teeth, not selected, but in the order of their extraction, mostly of first molars, from patients under twelve years of age, also a few specimens presenting marked peculiarities in root formation, but whose crowns are of normal type.

Of the first group I have removed the crowns so as to have the freest access to the bifurcations of the canals that the drills and instruments might have no hinderance in passing to and through the foramen. Of course no such presentation would occur in an ordinary operation in the mouth.

---

[1] Read before the Union Meeting, Springfield, Mass., October, 1889.

The results of my attempts to open these canals have been the breaking of drills in a few, puncturing at the side of the root in more, and succeeding in but a very small number of cases in passing at the proper point, and when doing so the *débris* would almost always be left outside in what would have been the "apical space." Another circumstance in connection with this drilling of the roots, which to me seems very important, and which I wish to emphasize, is that the amount of *heat* evolved in penetrating any of the smaller canals must do injury to the connective tissue, as it often forced me to let go the tooth with my fingers, although the engine was moving at an exceedingly slow speed. If the drill chips accumulated in the least, *heat* was the result, and these chips must be removed in some way to allow the work to go on. Till the foramen is pierced, this *débris* can only be lifted out by the drill, and that imperfectly.

You will please notice that several of these lower molars, although being from such young people, have exceedingly flat roots and very minute canals, with a division in one root into two distinct branches of a very small diameter and obscure entrance from the pulp chamber.

No instrument can penetrate these canals, and to attempt enlarging them is so difficult that the larger part of the crown must be sacrificed, and if much increase of diameter is attempted, penetration at the side of the root is sure to occur.

My second proposition is that nature is as fickle in a physiological and pathological sense, which I shall endeavor to establish by citations from my own practice.

1. A young lady when about fourteen years of age fell upon the ice and struck with great force upon the superior central incisors, the dentine became suffused with blood pigment, and when the case came into my hands, about three years later, both teeth were blue-black. After attempting to bleach, I excised the discolored crowns and replaced them with Logan crowns. On doing this the left central canal was found free to apex, but the right was solidly filled with secondary dentine, as far as I had need to drill. Neither tooth had given any trouble yet. Nature had been capricious.

2. A young man, about twenty, robust and a noted gymnast, came to me with swollen face, and left eye almost closed. On examination I found left central, a perfectly sound tooth as far as decay was concerned, abscessed and discharging pus copiously from numerous openings. The history of the case is as follows: He was employed in a bank, and was directed to prepare a large amount of silver coin for export, and in tying the parcels kept the end of the string between his teeth. The three-sided form of the package doubtless caused repeated jerks and jarring, the result upon the upper tooth being as I describe, the lower ones not being affected.

3. A lady in middle life presented a right superior molar, which had a fistula on the buccal side, with no cavity or discoloration observable. After she had borne the annoyance for some time, I removed the tooth and, upon examination, found complete ossification of the pulp and canals. (The tooth is now the property of Dr. Barrett, of Buffalo, who says it is the finest specimen he has ever seen.) Upon inquiry, I found this lady had been in the constant habit of cracking nuts and plum-pits upon every opportunity.

Again, a lower first molar was filled by Dr. Elliot, of this city, prior to 1855. I refilled it with same material, amalgam, a few years after; the pulp was then dead, as I presume it was at the first operation. No special effort was made to go beyond the pulp-chamber in cleaning or filling, creosote being applied. From that time till 1868 perfect peace reigned supreme.

In February of that year, patient became seriously ill in giving birth to a child without proper attendance, and very serious hemorrhage was the result, which was arrested by compress and plug. Within forty-eight hours the tooth described had the acute symptoms of alveolar abscess taking the usual course. (In this connection the question might be asked, Was this result one of those cases referred to by Dr. G. W. Black, where bacterial germs enter the circulation at a remote part and find fit conditions for multiplication?) Is it correet to say that that filling was a success by reason of the fact that for more than ten years it remained a useful member? I might weary you with additional evidence to uphold my proposition.

Cases are common of pivot teeth on the hickory peg, where the only treatment usually given was that of removing the pulp with a broach, enlarging the canal to the desired size, and then sending peg and crown to its resting-place to abide in peace for ten, twenty, and even thirty years. Not long since I removed four that had been in place for thirty-two years. Now, if this evidence is accepted, what ought the conclusion to be? Are there not limitations to our efforts to remove all the pulp from the roots?

I think it may safely be affirmed that only the single-rooted teeth can be well cleaned and filled, and even then much will depend on the locality of the cavity.

If it is declared *absolutely necessary*, the root to its apex *must*

be cleaned and filled to insure success, but even with this care many failures are inevitable.

Passing beyond the single-rooted teeth, difficulties multiply rapidly, such as flattened and bifurcated roots, tortuous, and curved in very minute points. If the cavities occur on posterior or labial face the sacrifice of a large part of the crown with a corresponding weakness must be the result to even reach the *entrances* to the canals. Often we have presented the second and third lower molars with pulp exposure, the cavity being below the margin of the gum either on the mesial or distal face, with a form of jaw that prevents a wide opening of the mouth, and on the superior jaw cavities of the lingual side.

Do the writers of the articles referred to intend to convey the idea that they remove all the *débris* to the apex of *each* of the several roots, and carry gold or cotton to their extremities?

Professor Truman, writing of the treatment of roots, uses these words: "The canal is thoroughly injected with peroxide of hydrogen as preliminary; this powerful oxidizer of organic matter prevents possible injury in cleaning out the *débris* of pulp tissue." Again, "The instruments used are passed through the alcohol flame," the "canal is then thoroughly washed with a certain solution, and when all odor of putrefaction ceases, the canal is generally considered to be ready for filling." Further on he says, "The canal is first closed at the apical foramen, at the shoulder, with either a small piece of cotton wet with carbolic, or better, if possible, with a small piece of gutta-percha, and chloride of zinc is then passed into the canal, to remain for several days." In the discussion of the paper some statements were made which, I think, would be modified under a cross-examination. I quote, and I believe fairly, for it would make this paper too long to make full extracts. Dr. L. G. Perry commends the paper in every particular. He uses gutta-percha points because it is possible to carry them to the end of the roots, and questions if it is not true, as claimed by Dr. Jack, that "the very small point of the canal can be filled as accurately with gold as with any other known means." Dr. J. Head "fills roots with cotton," but thinks there always is a strong probability that in molars the outer (?) portion of the apical foramen is unfilled. (Dr. Head seems to be the only one present at that discussion that hints at such possibilities.) Since this paper was prepared Dr. Head has an article in the October number of the INTERNATIONAL DENTAL JOURNAL referring to this same paper and discussion, in which he suggests that if they would perform the operations in teeth embedded in plaster and have them dissected afterwards, they would, probably, be convinced of the truth of his statements.

Dr. Dwinelle is very emphatic; he "fills the roots thoroughly and usually with gold." In fact, with hardly an exception, it seems to be unquestioned by the members present that Professor Truman's position is tenable: As I said at the beginning of this paper, I feel great delicacy in criticising such a body of leaders in dentistry whose papers and opinions are read with so much interest, but will not such statements mislead and deter our young and even some of our older members in their attempts to deal justly by themselves and towards their patients? When failure to save a tooth, after all their painstaking, meets them, will not the question arise, Who is to be compensated, patient or practitioner?

The conclusion of the matter seems to me to be, that averages rule in this as in many other things. We can only do what we can with each individual case, cultivating our discernment so as to *know* the best for each; remembering that there are the conditions of locality, as well as of disease, that will render our most intelligent and earnest efforts only failures, or, at best, but temporary successes, since circumstances may render our work valueless in the weeks that are to follow. Another matter that seems to me to have its objectionable feature is the number of agents recommended for use in septic conditions, with the maze of doubt as to which of the twenty-five or thirty will be the best; the changing treatment that must arise if the future does not bring the expected result; and when the effect desired does come, to which shall credit be given; these all seem to declare that it were far better to have some definite series of experiments made. My experiments seem to demonstrate that it is not often that the *débris* of the drill can be blown or washed out of the canals if the apex is not well open, and if open much of the contents will pass beyond the root, but in such cases septic treatment ought to correct any injurious effect. I indulge the hope that what I have here written may be received in the kind spirit in which it is given, and that each one may be stimulated to increase his successful averages.

Since writing the above article I have had some tubes prepared, partly filled with bone-dust, which I would like to see washed out or blown away by any of the means or instruments now used in the mouth. These tubes present a smoother and more perfect surface than those made in the canals, and if these cannot be cleaned how can the teeth?

